# Ferric citrate for the treatment of hyperphosphatemia and iron deficiency anaemia in patients with NDD-CKD: a systematic review and meta-analysis

**DOI:** 10.3389/fphar.2024.1285012

**Published:** 2024-03-07

**Authors:** Xueying Ding, Shujie Sun, Jinjin Zhang, Huifang Zhao, Fenglan Lun, Xuemin Liu, Yiwan Zhen, Jinping Dong, Jingliang Wu

**Affiliations:** Medical College, Weifang University of Science and Technology, Weifang, China

**Keywords:** chronic kidney disease, ferric citrate, hyperphosphatemia, iron deficiency anaemia, meta

## Abstract

**Background:** The application of ferric citrate therapy has yielded unexpected benefits in recent years for Chronic kidney disease patients suffering from hyperphosphatemia and iron deficiency -anaemia. Despite this, earlier research on the impact of ferric citrate on NDD-CKD has been contentious.

**Objective:** The goal of the meta-analysis is to evaluate the evidence regarding the advantages and dangers of ferric citrate for the treatment of hyperphosphatemia and iron deficiency anaemia in NDD-CKD patients.

**Methods:** Between the start of the study and June 2022, we searched PubMed, Embase, Cochrane, EBSCO, Scopus, Web of Science, Wan Fang Data, CNKI, and VIP databases for randomised controlled trials of iron citrate for hyperphosphatemia and anaemia in patients with NDD-CKD. For binary categorical data, risk ratios (OR) were employed, and for continuous variables, weighted mean differences The effect sizes for both count and measurement data were expressed using 95% confidence intervals

**Results:** The meta-analysis includes eight trials with a total of 1281 NDD-CKD patients. The phosphorus-lowering effect of ferric citrate was greater compared to the control group (WMD, −0.55, 95% CI, −0.81 to −0.28; I^2^ = 86%, *p* < 0.001). Calcium (WMD, 0.092; 95% CI, −0.051 to 0.234; *p* > 0.05; I^2^ = 61.9%), PTH (WMD, −0.10; 95% CI, −0.44 to 0.23; I^2^ = 75%, *p* > 0.05) and iFGF23 (WMD, −7.62; 95% CI, −21.18 to 5.94; I^2^ = 20%, *p* > 0.05) levels were not statistically different after ferric citrate treatment compared to control treatment. Furthermore, ferric citrate increased iron reserves and haemoglobin. The ferric citrate group had considerably greater levels than the controls. Ferric citrate, on the other hand, may raise the risk of constipation, diarrhoea, and nausea.

**Conclusion:** This meta-analysis found that ferric citrate had a beneficial effect in the treatment of NDD-CKD, particularly in reducing blood phosphorus levels when compared to a control intervention. It also shown that ferric citrate has a favourable effect on iron intake and anaemia management. In terms of safety, ferric citrate may increase the likelihood of gastrointestinal side effects.

## Introduction

Chronic kidney disease (CKD) is recognised as a major public health concern worldwide (Beng-Ongey et al., 2022). The global prevalence of CKD is estimated to be 13.4%, and while more than 5.9 million individuals require dialysis or functional kidney transplantation to stay alive, the great majority of CKD patients do not (Coresh et al., 2017). The most prevalent consequences in CKD patients are hyperphosphatemia and iron deficiency anaemia, both of which are independent risk factors for cardiovascular disease (Adejumo et al., 2015). In patients with CKD, the increasing reduction in glomerular filtration rate causes disruptions in calcium and phosphorus homeostasis, as well as reduced renal phosphorus excretion, which raises blood phosphorus levels (Khairallah et al., 2017). Dystrophic calcification, secondary hyperparathyroidism, and other mineral metabolism problems are also caused by hyperphosphatemia (Nakagawa and Komaba, 2018). Further research indicates that elevated phosphorus levels in early CKD increase the risk of anaemia (Tran et al., 2016). Anaemia causes decreased tissue oxygenation and aberrant cardiac compensatory mechanisms, which results in left ventricular hypertrophy and heart failure (Xu et al., 2017).

There are numerous commercially available phosphate binders used in CKD patients, but each has certain potential drawbacks (Nastou et al., 2014). Although effective, the calcium phosphate binders calcium acetate and calcium carbonate can cause vascular calcification (Cernaro et al., 2016). Furthermore, non-calcium phosphate binders are safer, but gastrointestinal issues and greater drug load are still clinical concerns (Wu-Wong et al., 2014). In terms of efficacy, safety, and cost, these medications are not comparable. Novel iron-containing phosphate binders may not only be useful in treating hyperphosphatemia, but they may also reduce the demand for intravenous iron and erythropoiesis-stimulating drugs in anaemia therapy for NDD-CKD patients (Nakanishi et al., 2016). New iron-containing phosphate binders (e.g., ferric citrate) may offer surprising benefits in terms of iron reserve replenishment and hyperphosphatemia improvement. Functioning as a phosphate binder, Ferric citrate dissociates iron ions in the gastrointestinal system. Insoluble iron phosphate is formed when these ions react with dietary phosphorus (Pai et al., 2016). Dietary phosphate is expelled rather than absorbed when this new molecule is excreted in the faeces (Erem and Razzaque, 2018). Iron salts diminish net intestinal phosphate absorption in rats, according to metabolic studies (Nakanishi et al., 2016). Not all iron ions dissociated from ferric citrate attach to phosphate as a source of elemental iron. The intestinal mucosa reduces some of them to ferrous iron via ferric reductase (Scheers et al., 2018). The iron becomes retained in the duodenum and is effectively reabsorbed into the systemic circulation, replenishing iron reserves as needed. Ferric citrate is an excellent treatment for hyperphosphatemia and iron insufficiency in NDD-CKD (Francis et al., 2019).

Ferric citrate as a phosphate binder with meals has been proven by studies to be efficient in lowering FGF23 and boosting iron parameters in NDD-CKD (Ogata et al., 2022). Despite ferric citrate’s capacity to bind phosphate in the gastrointestinal system, evidence for therapeutic applicability is lacking. The recently updated Kidney Disease Improving Global Outcomes (KDIGO) guidelines emphasise that lowering serum phosphate with ferric citrate improves the prognosis of CKD patients, but there are not enough clinical trials with NDD-CKD patients, raising concerns about whether ferric citrate is effective and safe in this population (Inker et al., 2014; Bazeley and Wish, 2022). Ferric citrate was approved as a phosphorus-reducing medication for dialysis patients with CKD by the US Food and Drug Administration (FDA) in 2014 (Shah et al., 2015). Ferric citrate was authorised as an alternate therapy for iron deficiency anaemia in individuals with NDD-CKD in 2017 (Fishbane et al., 2017). However, because ferric citrate is a new medicine, post-marketing monitoring studies have yet to confirm its therapeutic effectiveness and safety in patients with NDD-CKD (Inker et al., 2014).

Although the effectiveness and safety of ferric citrate have been thoroughly reviewed and meta-analysed, the majority of studies have only included CKD patients on dialysis. The best management approach for NDD-CKD serum phosphate retention and iron-deficient anaemia is unknown. As a result, the goal of this meta-analysis was to evaluate data from trials on ferric citrate’s ability to reduce blood phosphorus, iron intake, anaemia therapy, and adverse medication events in order to give doctors recommendations on ferric citrate usage in NDD-CKD patients.

## Materials and methods

### Literature search

For publishing systematic reviews and meta-analyses, we followed the PRISMA declaration and the Cochrane Handbook criteria. PubMed, Scopus, Cochrane, EBSCO, Embase, Web of Science, the Chinese National Knowledge Infrastructure, Wan Fang Data, and the VIP Chinese Journal Service platform were among the search databases used. The deadline for doing a literature search is June 2022. Our search strategy includes the keywords “Chronic Kidney Disease” or “Kidney Insufficiencies, Chronic” or “Chronic Renal Insufficiencies” or “Renal Disease, Chronic” combined with “Ferric Citrate” or “JTT-751″or “Iron (III) Citrate” or “Ferric Citrate Hydrate” or “Ferric Citrate Anhydrous” or “Ferric-Citric Acid”. We used the terms “all fields,” “titles,” and “keywords” to search PubMed and other databases.

### Eligibility criteria

#### Types of participants

(1) If the study was a randomised controlled trial, it qualified for inclusion. (2) Patients with CKD who are not on dialysis. (3) Hyperphosphatemia (or) iron insufficiency (or both). (4) Participants were followed for at least 12 weeks after randomisation.

#### Intervention measures

The placebo, no study medicine, normal care, or alternative therapy for iron deficient anaemia was given to the control group. The ferric citrate therapy was given to the experimental group.

#### Outcomes

The major result measured was the difference in biochemical markers such as serum phosphate, haemoglobin, ferritin, and transferrin saturation (TSAT) across groups. Differences in calcium, parathyroid hormone (PTH), intact fibroblast growth factor 23 (iFGF23), and unpleasant effects (constipation, diarrhoea, stomach discomfort, nausea, and stool discolouration) were also investigated as secondary outcomes.

#### Exclusion criteria

Any study that satisfies the following criteria must be excluded from meta-analyses: (1) Literature with insufficient data reporting. (2) Studies with inadequate data to allow for interpretation of findings. (3) Non-ferric citrate treated or dialysis CKD patients are included in the literature. (4) Reviews, *in vitro* research, case reports, conference papers, and animal trial studies.

### Data extraction and management

Two researchers who independently researched the literature collected and cross-checked the data. If conflicts arose during the screening of the literature, they should be addressed through discussion or negotiation with a third reviewer. All included literature was classified and analysed using office software, including research author, publication year, study design, control drug, drug dose, follow-up period, patient numbers, patient gender, CKD stage, blood phosphorus, and iron levels at baseline. If any information is missing from the study, the original author is contacted by phone or email.

#### Literature quality assessment

The risk of bias in the collected literature was evaluated using the procedures outlined in the Cochrane Handbook for Systematic Reviews of Interventions 5.1.0. The randomisation process, the allocation concealment design, the blinding methods utilised, the reporting of research results, the existence of additional sources of bias, and any selective reporting of research results are among the contents evaluated. The findings are as follows: “Yes” means accurate methodology or complete data, suggesting a low risk of bias; “unclear” means a medium risk of bias; and “No” means erroneous methodology or incomplete data, indicating a high risk of prejudice. Finally, data was imported into Revman 5.4 software, and the risk of bias assessment plots were exported.

#### Statistical analysis

Data analysis was carried out using the Revman 5.3 programme. In the meta-analysis, we estimated the combined relative risk (RR) for categorical variables. We estimated the mean difference (MD) for continuous variables. The 95% confidence intervals (CI) were used to express the data impacts. The I^2^ index was used to measure statistical heterogeneity, with low heterogeneity defined as I^2^ < 50%. Furthermore, to confirm that dosage considerations did not alter the substance treatment effect, we ran further subgroup analyses based on different medication doses (≥5 g, <5 g). If there was evidence that a study differed significantly from other studies in terms of methodology or findings, sensitivity analyses were then carried out exclude these studies from the meta-analysis. Egger’s quantitative test of *p* > 0.05 was considered statistically significant to assess for existing publication bias. To establish whether there was any asymmetry, funnel plots were used to undertake a qualitative evaluation. Making funnel plots with “STATA” (version 14) software.

## Results

### Search results

**FIGURE 1:**
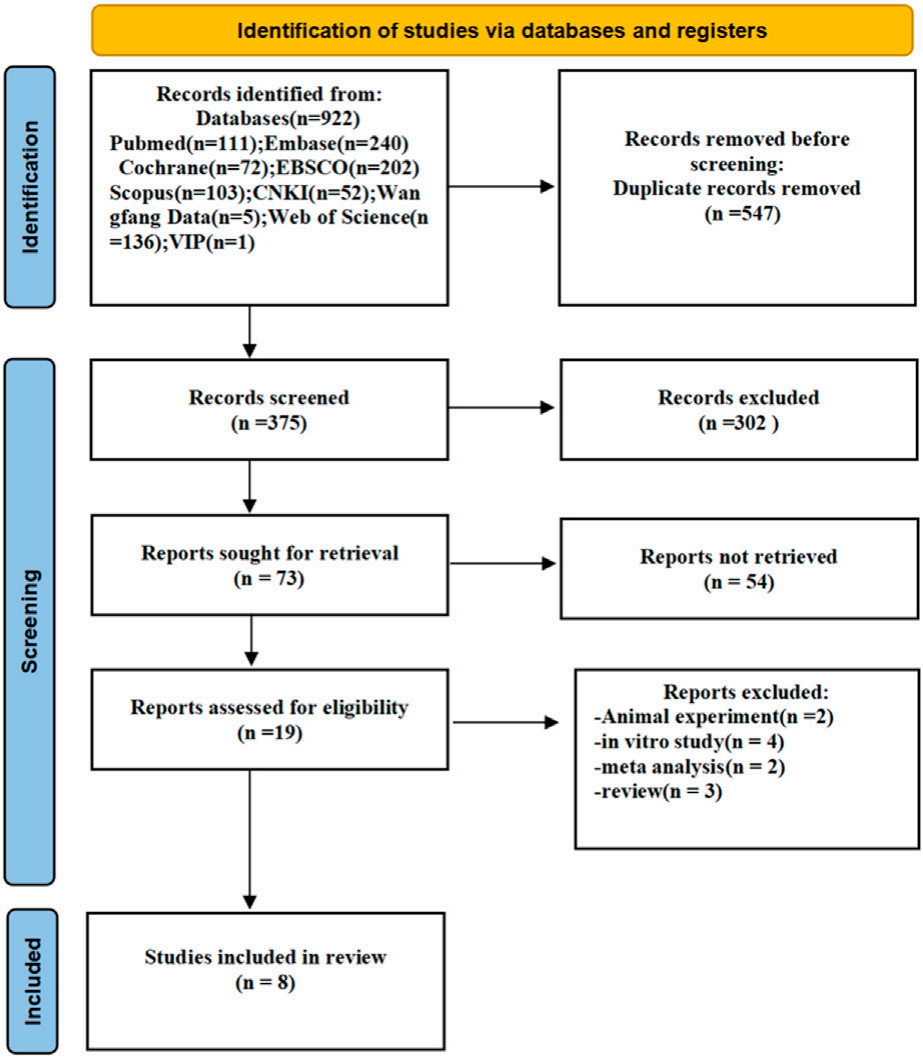
Flow chart of the study selection process.

The search strategy is mostly demonstrated in Supplementary Material S1. The PRISMA checklist may be found in Supplementary Material S2. Figure 1 depicts the meta-analysis study selection diagram. The original database search yielded 922 citations (PubMed: 111; Web of Science: 136; Scopus: 103; Cochrane: 72; EBSCO: 202; Embase: 240; CNKI: 52; Wan fang Data: 5; VIP Chinese Journal Service Platform: 1), 547 of which were removed from the duplicate option. The remaining 375 trials were subjected to further title and abstract screening. The 302 articles were rejected for the following reasons. The remaining 73 trials’ full texts were checked again, and a total of 8 studies are appropriate for statistical analysis.

### Study characteristics and quality assessment

The features of the eligible studies are described in Table 1. There were 1,281 individuals with NDD-CKD in eight studies (sample size range: 23–234 patients; follow-up range: 12–36 weeks). There were eight controlled studies, six of which were double-blind and two of which were open-label. The majority of these investigations took place in three countries: China, Japan, and the United States. The control group was given ferrous sulphate treatment, normal care, placebo therapy, or no treatment at all. There were no significant variations in baseline information between the experimental and control groups in terms of patient inclusion criteria.

**TABLE 1:** Characteristics of included studies.

Study (Year)	Country	Study type	Follow-up (weeks)	Ferric citrate (daily/dose) (mean)	C (g)ontrols (daily/dose) (mean)	Number of patients	CKD stage	Baseline serum phosphorus (mg/dL) (mean)	Baseline iron related parameters	Outcomes
Iguchi 2017	Japan	Open-label, pilot, single-center randomized	12	0.75	None	23	3–5	3.6 mg/dL	TSAT ≤20%, ferritin ≤100 mcg/L	2 3 4 5 6 7 8
Fishbane 2017	American	Randomized, double-blind, placebo-controlled, multicenter	16	5.0	Placebo 5.1 g	234	3–5	4.4 mg/dL	TSAT ≤25%, ferritin ≤200 mcg/L	1 2 3 4 6 7 9 10 11 12 13 14
Yokoyama 2014	Japan	Randomized, double-blind, placebo-controlled, multicenter	12	3.5	Placebo	90	3–5	5.6 mg/dL	TSAT ≤50%, ferritin ≤500 mcg/L	1 2 3 4 5 6 7 8 9 10 11
Chertow 2017	American	Randomized, multicenter, double-blind, placebo-controlled	12	5.4	Placebo 5.4 g	380	3–5	4.3 mg/dL	TSAT <30%, ferritin <674 mcg/L	1 6 7 8 9 10 11 12 13 14
Block 2015	American	Randomized, double-blind, placebo-controlled, multicenter	12	5.1	Placebo 5.2 g	149	3–5	4.6 mg/dL	TSAT ≤30%, ferritin ≤300 mcg/L	9 10 11 12 13 14
Block 2019	American	Open-label, pilot, single-center randomized	36	5.0	Usual care	195	4–5 (advanced CKD)	4.5 mg/dL	TSAT <55%	1 2 3 5 6 7 8
Womack 2020	American	Randomized, double-blind	12	2.0	Ferrous sulfate 0.325 g	60	3–4	3.4 mg/dL	TSAT ≤20%, ferritin ≤100 ng/mL	7 8 9 10 11
Lin 2021	China	Randomized control trial	12	3.0	None	150	4–5 (advanced CKD)	-	TSAT <55%, ferritin ≤500 mcg/L	6

Note: 1. Serum phosphorus; 2. Calcium; 3. PTH; 4. iFGF23 5. Iron; 6. Haemoglobin; 7. Ferritin; 8. TSAT; 9. Constipation; 10. Diarrhea; 11. Nausea; 12. Abdominal pain; 13. Abdominal discomfort; 14. Fecal discoloration.

**FIGURE 2:**
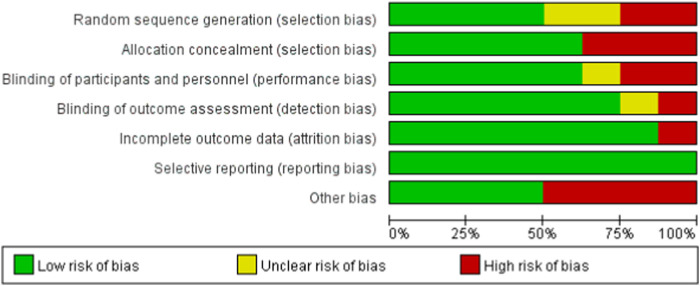
Risk of bias graph.

**FIGURE 3:**
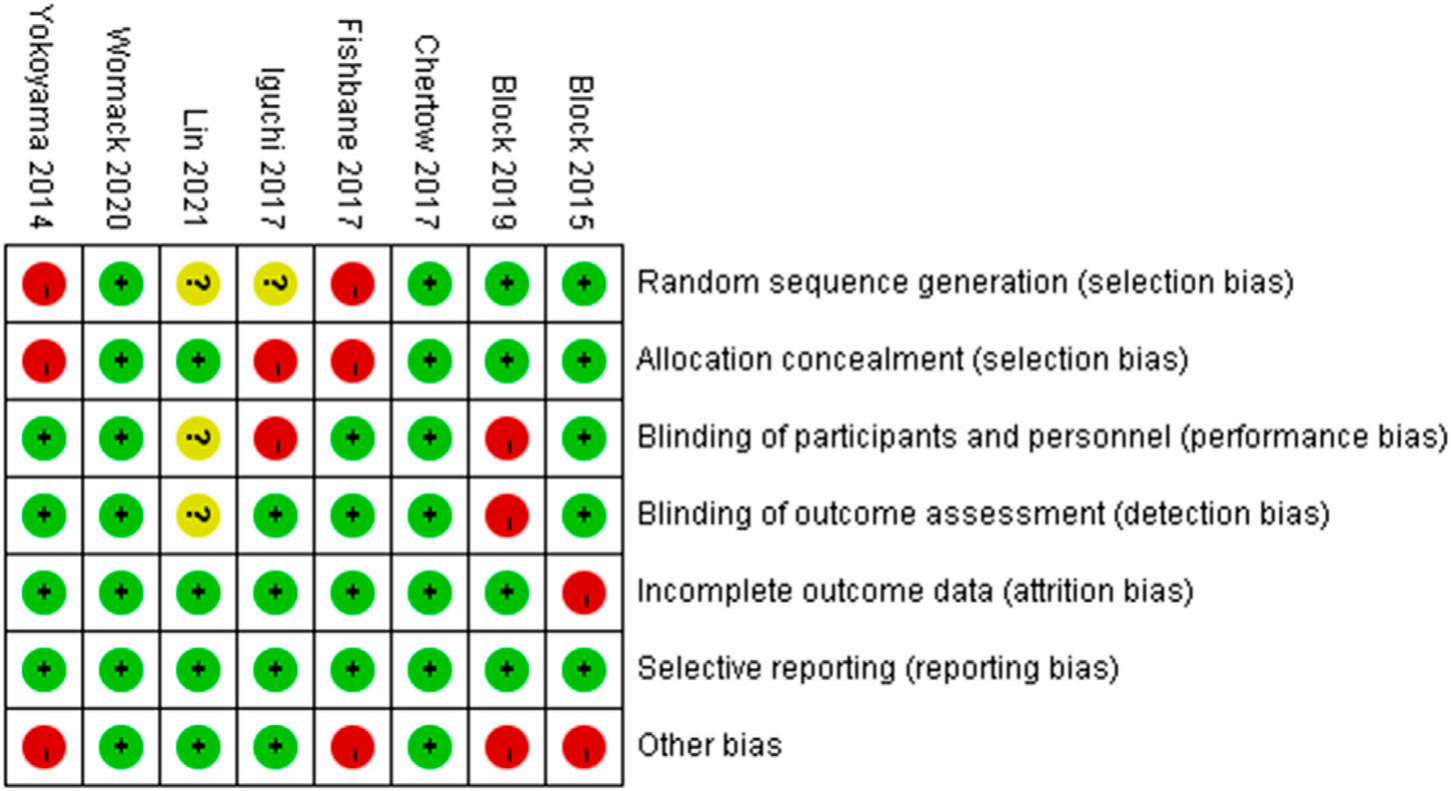
Risk of bias summary.

Figures 2, 3 illustrate the risk of bias assessment. In addition, blinding of participants and investigators to the assigned intervention was reported to be low in 63% (5/8) of trials, while outcome assessor blinding was reported to be low in 88% (7/8) of trials. Reasons and figures for withdrawal and abandonment were mentioned in each article. The majority of the studies were based on low-bias analysis.

### Meta-analysis results

#### The variations of phosphorus, calcium, PTH, and iFGF23 levels in serum

**FIGURE 4:**
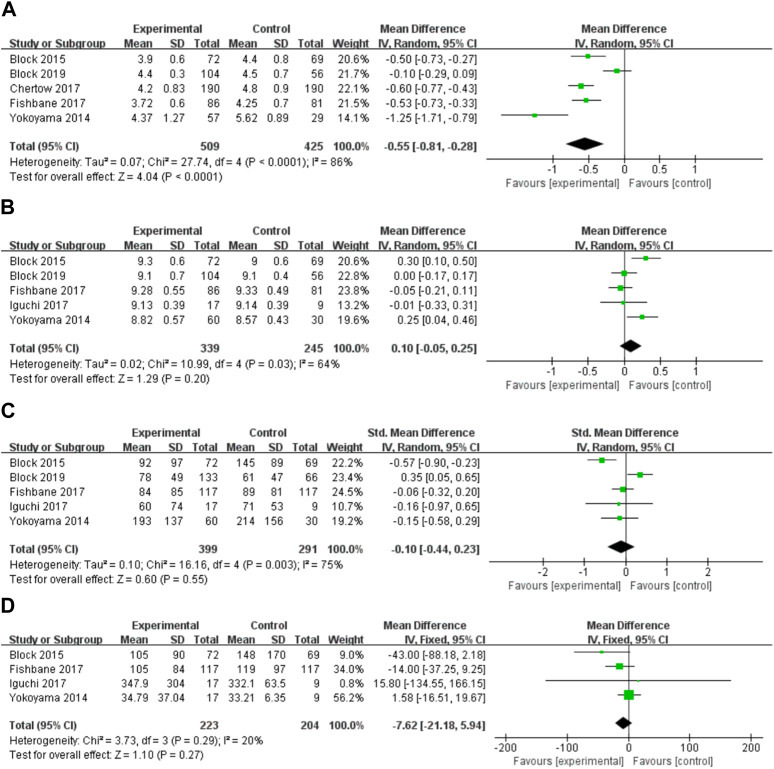
Forest plot of the correlation between hyperphosphatemia and ferric citrate in NDD-CKD patients: **(A)** Changes in serum phosphorus; **(B)** Changes in serum calcium; **(C)** Changes in PTH; **(D)** Changes in iFGF23.

**FIGURE 5:**
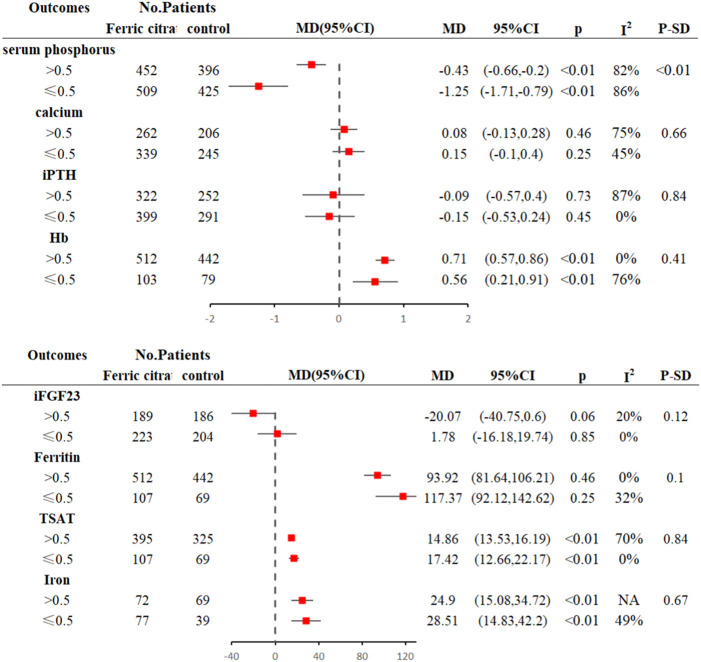
Subgroup analysis of daily doses of ferric citrate.

Ferric citrate substantially lowered blood phosphate levels compared to placebo and usual care in five (Yokoyama et al., 2014; Block et al., 2015; Chertow et al., 2017; Fishbane et al., 2017; Block et al., 2019) investigations (WMD, −0.55, 95% CI, −0.81 to −0.28; I^2^ = 86%, *p* < 0.001; Figure 4A). Because the results were highly heterogeneous, a random effects model was employed. Individual study order sensitivity analysis demonstrated no discernible influence on total combined WMD, supporting the validity and plausibility of combined WMD. Five trials (Yokoyama et al., 2014; Block et al., 2015; Fishbane et al., 2017; Block et al., 2019) found no significant impact of iron citrate on blood calcium concentrations compared to placebo or usual care (WMD, 0.25; 95% CI, −0.04 to 0.46; I^2^ = 64%, *p* > 0.05; Figure 4B). PTH outcomes were reported in five studies (Yokoyama et al., 2014; Block et al., 2015; Fishbane et al., 2017; Block et al., 2019), and meta-analysis revealed no statistically significant differences between the control (placebo or usual care) and ferric citrate groups, indicating that ferric citrate did not cause changes in PTH levels (WMD, −0.10; 95% CI, −0.44 to 0.23; I^2^ = 75%, *p* > 0.05; Figure 4C). The random effects model was adopted in this investigation because of the considerable heterogeneity. A meta-analysis of four investigations on iFGF23 (Yokoyama et al., 2014; Block et al., 2015; Fishbane et al., 2017; Block et al., 2019) found no significant difference between treatment and control groups, indicating that the impact of ferric citrate on iFGF23 was uncertain (WMD, −7.62; 95% CI, −21.18 to 5.94; I^2^ = 20%, *p* > 0.05; Figure 4D). None of the measures showed significant changes throughout the dosage subgroup analysis, confirming the robustness of our findings (Figure 5).

#### Anaemia and iron laboratory parameters affected by ferric citrate

**FIGURE 6:**
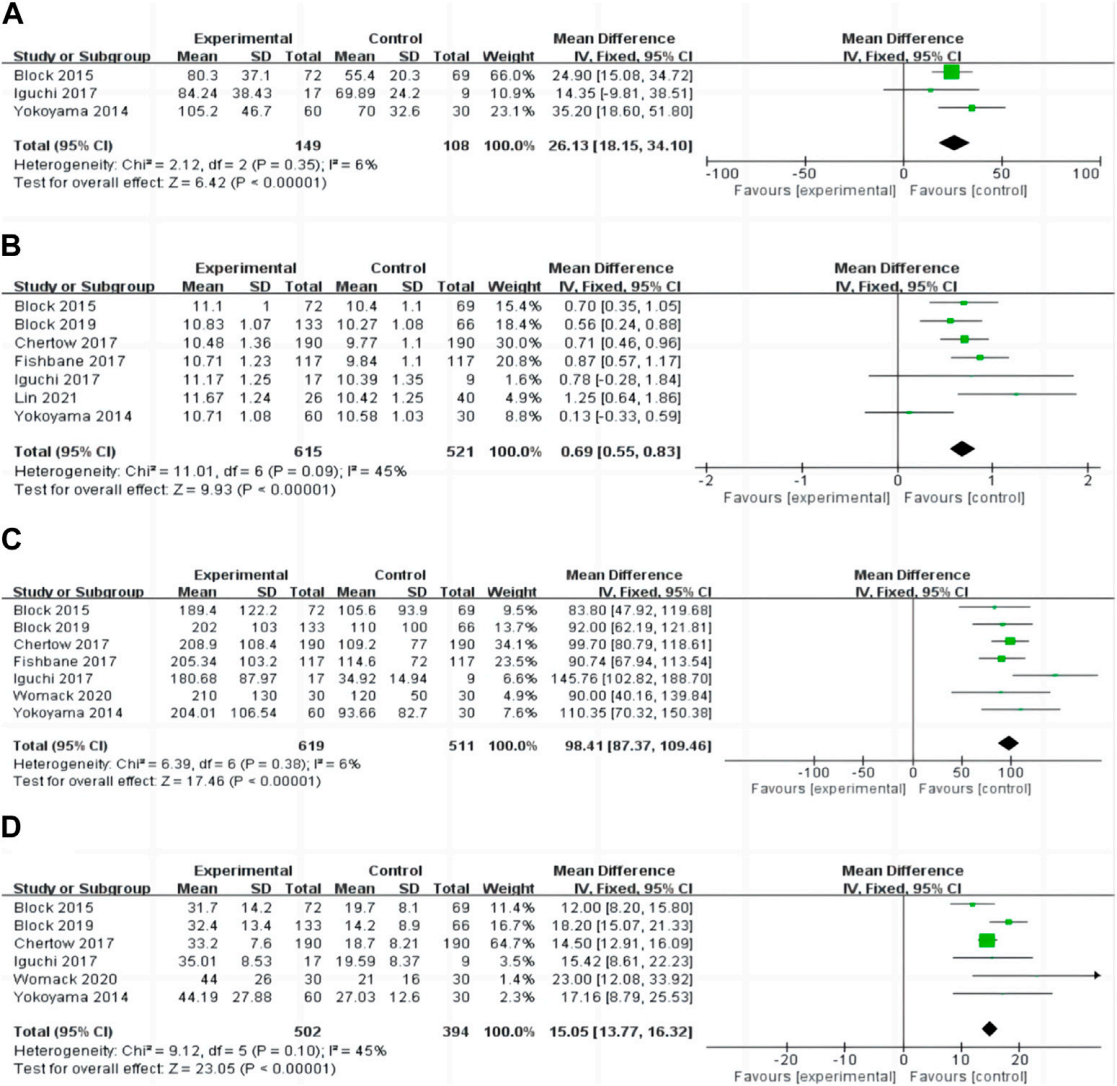
Forest plot of the correlation between iron deficiency anaemia and ferric citrate in NDD-CKD patients: **(A)** Changes in iron; **(B)** Changes in haemoglobin; **(C)** Changes in ferritin; **(D)** Changes in TSAT.

We evaluated the effect of ferric citrate on additional lab findings, including iron, haemoglobin, ferritin, and TSAT, to study the potential advantages of ferric citrate in treating iron-deficient anaemia in NDD-CKD patients. Three studies (Yokoyama et al., 2014; Block et al., 2015; Iguchi et al., 2018) found that post-treatment iron levels in NDD-CKD patients were significantly higher in the ferric citrate group than in the control group (WMD, 26.13; 95% confidence interval, 18.15 to 34.10; I^2^ = 6%, *p* < 0.001; Figure 6A). Seven studies (Yokoyama et al., 2014; Block et al., 2015; Chertow et al., 2017; Fishbane et al., 2017; Iguchi et al., 2018; Block et al., 2019; Lin et al., 2021) reported haemoglobin alterations and the meta-analysis revealed that all patients in the ferric citrate therapy group had substantially higher haemoglobin levels than control patients (WMD, 0.69; 95% CI, 0.55 to 0.83; I^2^ = 45%, *p* < 0.001; Figure 6B). In the ferritin and TSAT meta-analysis, ferric citrate was effective in increasing serum ferritin and TSAT levels (WMD, 98.41; 95% CI, 87.37 to 109.46; I^2^ = 6%, *p* < 0.001; Figure 6C), (WMD, 15.05; 95% CI, 13.77 to 16.32; I^2^ = 45%, *P* 0.001; Figure 6D). Laboratory measures of anaemia and iron showed no change in the dose subgroup analyses, indicating reliable results. (Figure 5).

#### Adverse event of ferric citrate

**FIGURE 7:**
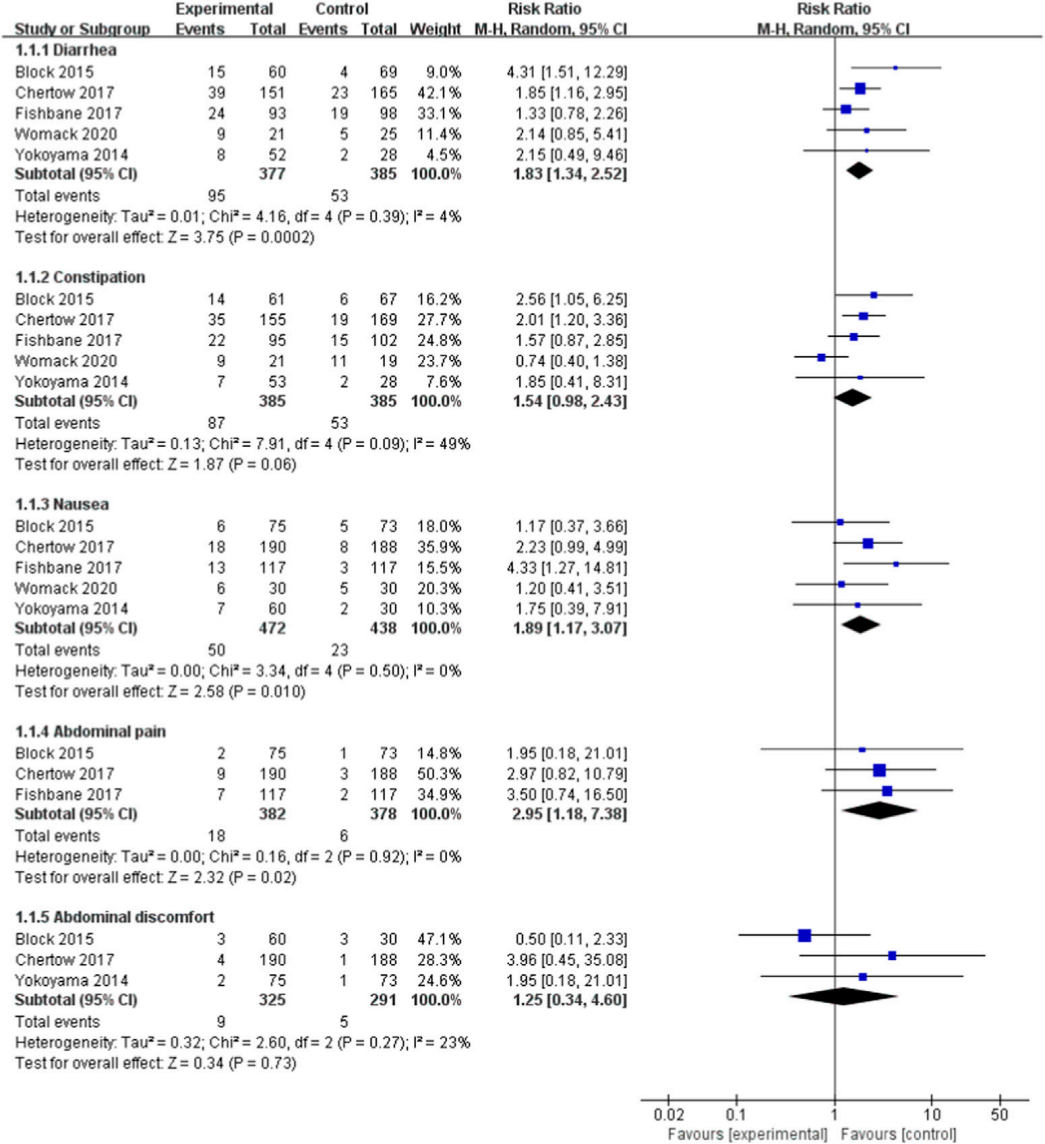
The forest plot shows adverse event.

Five (Yokoyama et al., 2014; Block et al., 2015; Chertow et al., 2017; Fishbane et al., 2017; Womack et al., 2020) studies, including 910 NDD-CKD patients, found harmful effects of ferric citrate. For each category of adverse event, we summarise the findings of our risk analysis (Figure 7). Diarrhoea, constipation, nausea, stomach pain, and discomfort were the most prevalent side effects. According to the pooled risk analysis, patients who received ferric citrate had a considerably increased probability of developing diarrhoea, nausea, and stomach discomfort than patients in the comparison group, with RRs of (RR, 1.83; 95% CI, 1.3 4 to 2.52; I^2^ = 4%, *p* < 0.001), (RR, 1.89; 95% CI, 1.71 to 3.07; I^2^ = 0%, *p* = 0.01) and (RR, 2.95; 95% CI, 1.18 to 7.38; I^2^ = 0%, *p* = 0.02).

#### Publication bias

**FIGURE 8:**
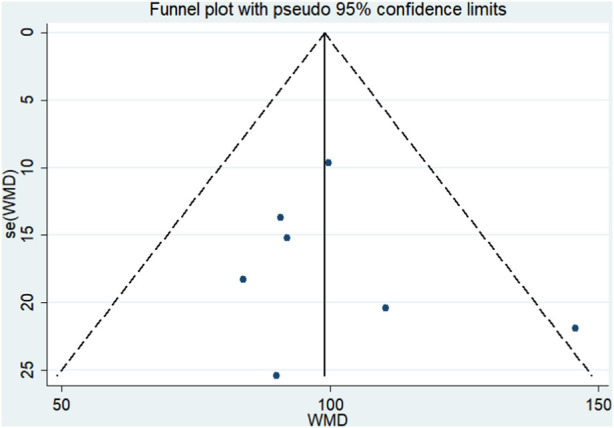
Funnel plot of ferritin.

A funnel plot was created to examine publication bias for the indicators with the most included papers. Ferritin levels in NDD-CKD patients revealed a clear symmetric funnel plot (Figure 8). Egger’s findings imply that the study bias was small (*p* = 0.57).

## Discussion

The efficiency of ferric citrate in managing key laboratory measures of hyperphosphatemia in NDD-CKD patients was studied in this meta-analysis. To investigate the effect of ferric citrate on iron replenishment and anaemia alleviation, data on iron-anaemia profiles, including haemoglobin levels and iron reserves, were also obtained. The safety of ferric citrate was investigated more thoroughly by summarising adverse occurrences associated with it. In this comprehensive review and meta-analysis, ferric citrate was shown to be more effective than placebo or standard therapy at controlling phosphorus levels in patients with NDD-CKD. Other biochemical indicators of blood calcium and mineral metabolism (e.g., PTH and iFGF23) were not affected by ferric citrate. The study also discovered that ferric citrate is helpful in replenishing iron reserves and partially correcting anaemia. Further research discovered that ferric citrate therapy dramatically boosted iron stores, haemoglobin, ferritin, and TSAT levels. In terms of safety, we discovered that using ferric citrate marginally raised the likelihood of gastrointestinal side effects.

Patients with CKD are more likely to have various problems when their renal function deteriorates (Baluarte, 2017). Because the kidneys play an important role in excreting phosphorus and maintaining homeostasis, hyperphosphatemia is a risk factor for cardiovascular disease and death. Abnormal phosphorus levels in the blood alter serum electrolyte concentrations as well as PTH balance, resulting in bone remodelling problems and vascular calcification (Wang et al., 2015). Our systematic review found that ferric citrate was better than placebo or conventional standard therapy in lowering blood phosphorus levels in NDD-CKD patients, which is consistent with a previous meta-analysis (Choi et al., 2021). Ferric citrate was initially authorised for lowering blood phosphorus levels in CKD patients (Lewis et al., 2015). However, therapeutic effects for treating hyperphosphatemia in NDD-CKD patients are largely unknown. To reduce blood phosphorus levels, KDIGO clinical recommendations now advocate reducing phosphate intake in the diet and utilising phosphorus-lowering medicines to minimise intestinal absorption of phosphorus (Dhillon-Jhattu and Sprague, 2018). However, because many patients are already on nutritionally restricted diets, it may be difficult for them to lower their dietary phosphate consumption. Phosphate binders are considered the first-line treatment for hyperphosphatemia in these individuals. Insoluble iron phosphate is formed when iron ions in ferric citrate complexes bond to ingested phosphorus. Dietary phosphate is excreted rather than absorbed by excreting this new compound (Dhillon-Jhattu and Sprague, 2018). As a result, ferric citrate is efficient in inhibiting dietary phosphate absorption into the body’s circulation.

The study’s findings revealed no statistically significant change in blood calcium levels between the ferric citrate and control groups. The ferric citrate group showed an increasing tendency in calcium alterations, but the results were not statistically significant. None of the included trials reported any occurrences of hypercalcemia linked with iron citrate. According to certain research, ferric citrate may also enhance intestine-free calcium utilisation and produce calcium homeostasis abnormalities (Negri and Ureña Torres, 2015). Although newer calcium-free treatments, such as sevelamer, are efficacious and have a low incidence of hypercalcemia, their high cost, pill load, and gastrointestinal side effects might cause patient compliance issues (Palmer et al., 2016; Schumacher et al., 2019). As a result, finding a safe, effective, and low-cost phosphate binder for chronic renal disease patients with hyperphosphatemia would be advantageous. Second, except for better biochemical results in serum phosphate levels, our meta-analysis in an NDD-CKD population found no documented advantage of ferric citrate on PTH and iFGF23. The findings of this meta-analysis contradict earlier research suggesting that ferric citrate therapy decreases iFGF23 levels over time (Choi et al., 2021). However, the current study’s lack of conclusive statistical significance might be ascribed to the small patient sample size or the study’s unusually short length. As a result, there was no statistically significant difference between the treatment and control groups. Given that the majority of studies endure between three and 9 months, it may take many years to see any possible benefit in terms of persistent declines in PTH or iFGF23. The potential advantages of clinical outcomes must be evaluated in a two to 3-year randomised controlled study.

There was substantial variation between trials examining changes in serum phosphorus, calcium, PTH, and iFCG23 levels following therapy. As a result, a random effects model was adopted to evaluate mixed effects. For outcomes with a significant degree of heterogeneity, sensitivity analysis was carried out. Significant heterogeneity may be connected with the registered patients’ varied baseline characteristics. The age distribution of the patients participating in this study, for example, was diverse, with the majority of them being middle-aged individuals. Furthermore, individuals with NDD-CKD at various phases, such as intermediate and end-stage renal disease, were included.

Iron deficiency anaemia is a typical consequence of CKD that develops over time as the illness progresses. Anaemia in CKD patients is exacerbated by iron shortage, iron malabsorption, uneven iron distribution, and decreased red blood cell lifetime owing to uraemia (Rivera et al., 2017; Batchelor et al., 2020). Because there is a dearth of evidence for treating NDD-CKD patients compared to CKD patients on dialysis, this patient population has clinical challenges in terms of anaemia management. KDIGO guidelines recommend that for NDD-CKD patients who require iron supplementation, the route of iron administration be chosen based on the severity of the iron deficiency, the availability of intravenous access, the side effects of oral or intravenous iron therapy, patient compliance, and cost (Rivera et al., 2017). As a result, healthcare practitioners are reconsidering oral iron treatment, particularly for those with NDD-CKD. According to one recent study, one-third of patients received ferrous sulphate orally, although ferric citrate absorbs elemental iron three to four times better than ferrous sulphate (Cirillo et al., 2021). Several studies show that ferric citrate is poorly absorbed in the gastrointestinal system, although citrate in the intestinal lumen partially increases iron absorption and has enough iron supplementation action to considerably reduce ESA usage in NDD-CKD patients (Negri and Ureña Torres, 2015; Yagil et al., 2015). Our findings are consistent with previous research on dialysis CKD patients. According to the findings of this meta-analysis, ferric citrate increased iron consumption as well as haemoglobin, ferritin, and TSAT levels. Notably, recent research on phosphorus homeostasis molecular processes shows a link between hyperphosphatemia and iron insufficiency via the regulating hormone iFGF23. In NDD-CKD patients, ferric citrate taken with meals lowered iFGF23 while increasing iron parameters (Iida et al., 2020). Although the current investigation found that iron citrate had no effect on iFGF23, its efficacy in treating iron deficiency and reducing blood phosphorus levels in NDD-CKD patients is undeniable.

The meta-analysis discovered no major or unexpected safety risks. In comparison to the control group, ferric citrate induced more adverse effects, including constipation, diarrhoea, nausea, faecal discolouration, stomach pain, and abdominal discomfort. Biruete et al. (Biruete et al., 2020) further confirm that non-calcium-based phosphate binders are mostly associated with gastrointestinal side effects. Fortunately, extensive research has indicated that patients managed diarrhoea, stomach pain, and discomfort well. This issue was also temporary and was not disruptive to treatment. Furthermore, Yokoyama et al. (Yokoyama et al., 2014) discovered a dose-dependent incidence of diarrhoea with ferric citrate therapy. Patients receiving 6 g per day showed a rather high incidence, whereas those receiving 1.5 and 3 g had an incidence comparable to the placebo group. Notably, the frequency of all gastrointestinal adverse effects reduced with time despite higher ferric citrate dosages. Patients should also be reminded that alterations in faecal features should be reviewed with their healthcare practitioner after they have had therapy to prevent disguising other gastrointestinal disorders (for example, black stools or blood in the stool).

Serious side effects such as infection and hospitalisation were not recorded in the included trials. Based on the findings of the current investigation, iron citrate appears to be a viable therapy for hyperphosphatemia and iron-deficient anaemia in individuals with NDD-CKD. Furthermore, ferric citrate has the potential to lower medication prices and burdens. The drug’s effectiveness is unaffected by gastrointestinal tract pH or the concurrent administration of antacids or H2 receptor antagonists (Segregur et al., 2019). There is, however, no compelling scientific evidence that phosphate management improves patient clinical outcomes, including all-cause mortality and cardiovascular issues. Several prospective studies are now registered on ClinicalTrials.gov to investigate the function of phosphate binding in enhancing clinical outcomes. To fully analyse the advantages and dangers of this medication therapy, we urge future studies on the long-term efficacy and influence of ferric citrate on clinical outcomes, such as secondary complication prevention, drug burden reduction, safety, and cost savings (Wu et al., 2017).

### Limitation

This study has a few limitations: The trials employed a variety of pharmacological dosages and treatment durations, and the research follow-up periods were often brief. Second, a substantial degree of heterogeneity was discovered in some outcomes. Individual studies gradually raised the ferric citrate dosage, whilst others utilised several doses or stable amounts throughout therapy. These dosages and concurrent drugs may contribute to the observed high degree of variability. Our findings, on the other hand, confirm and validate the role of ferric citrate in the treatment of hyperphosphatemia and iron deficiency anaemia in patients with NDD-CKD, and this study on the impact of ferric citrate on these outcomes may provide insight into the efficacy and safety of ferric citrate on clinically important outcomes.

## Conclusion

This systematic review is an important addition since it summarises the existing evidence on ferric citrate therapies in NDD-CKD and adds to the existing clinical trial data. Ferric citrate, according to our meta-analysis, is effective enough to be considered for the treatment of hyperphosphatemia and iron-deficient anaemia in NDD-CKD. Ferric citrate lowered blood phosphorus levels more effectively than controls in individuals with NDD-CKD and showed equivalent efficiency in managing anaemia. The most common side effects of iron citrate were diarrhoea, constipation, and nausea.

## References

[B1] Adejumo O. A. Okaka E. I. Madumezia G. Okwuonu C. G. Ojogwu L. I. (2015). Assessment of some cardiovascular risk factors in predialysis chronic kidney disease patients in Southern Nigeria. Niger. Med. J. J. Niger. Med. Assoc. 56 (6), 394–399. 10.4103/0300-1652.171616 PMC4743288 26903696

[B2] Baluarte J. H. (2017). Neurological complications of renal disease. Seminars Pediatr. neurology 24 (1), 25–32. 10.1016/j.spen.2016.12.004 28779862

[B3] Batchelor E. K. Kapitsinou P. Pergola P. E. Kovesdy C. P. Jalal D. I. (2020). Iron deficiency in chronic kidney disease: updates on pathophysiology, diagnosis, and treatment. J. Am. Soc. Nephrol. JASN 31 (3), 456–468. 10.1681/ASN.2019020213 32041774 PMC7062209

[B4] Bazeley J. W. Wish J. B. (2022). Recent and emerging therapies for iron deficiency in anemia of CKD: a review. Am. J. kidney Dis. official J. Natl. Kidney Found. 79 (6), 868–876. 10.1053/j.ajkd.2021.09.017 34758368

[B5] Beng-Ongey H. Robinson J. S. Moxey-Mims M. (2022). Chronic kidney disease emerging trends in children and what to do about it. J. Natl. Med. Assoc. 114 (3s2), S50–s55. 10.1016/j.jnma.2022.05.002 35660045

[B6] Biruete A. Hill Gallant K. M. Lindemann S. R. Wiese G. N. Chen N. X. Moe S. M. (2020). Phosphate binders and nonphosphate effects in the gastrointestinal tract. J. Ren. Nutr. official J. Counc. Ren. Nutr. Natl. Kidney Found. 30 (1), 4–10. 10.1053/j.jrn.2019.01.004 PMC6722023 30846238

[B7] Block G. A. Block M. S. Smits G. Mehta R. Isakova T. Wolf M. (2019). A pilot randomized trial of ferric citrate coordination complex for the treatment of advanced CKD. J. Am. Soc. Nephrol. JASN 30 (8), 1495–1504. 10.1681/ASN.2018101016 31278194 PMC6683712

[B8] Block G. A. Fishbane S. Rodriguez M. Smits G. Shemesh S. Pergola P. E. (2015). A 12-week, double-blind, placebo-controlled trial of ferric citrate for the treatment of iron deficiency anemia and reduction of serum phosphate in patients with CKD Stages 3-5. Am. J. kidney Dis. official J. Natl. Kidney Found. 65 (5), 728–736. 10.1053/j.ajkd.2014.10.014 25468387

[B9] Cernaro V. Santoro D. Lacquaniti A. Costantino G. Visconti L. Buemi A. (2016). Phosphate binders for the treatment of chronic kidney disease: role of iron oxyhydroxide. Int. J. Nephrol. renovascular Dis. 9, 11–19. 10.2147/IJNRD.S78040 PMC4749089 26893577

[B10] Chertow G. M. Block G. A. Neylan J. F. Pergola P. E. Uhlig K. Fishbane S. (2017). Safety and efficacy of ferric citrate in patients with nondialysis-dependent chronic kidney disease. PloS one 12 (11), e0188712. 10.1371/journal.pone.0188712 29186198 PMC5706696

[B11] Choi Y. J. Noh Y. Shin S. (2021). Ferric citrate in the management of hyperphosphataemia and iron deficiency anaemia: a meta-analysis in patients with chronic kidney disease. Br. J. Clin. Pharmacol. 87 (2), 414–426. 10.1111/bcp.14396 32470149

[B12] Cirillo L. Somma C. Allinovi M. Bagalà A. Ferro G. Di Marcantonio E. (2021). Ferric carboxymaltose vs. ferrous sulfate for the treatment of anemia in advanced chronic kidney disease: an observational retrospective study and cost analysis. Sci. Rep. 11 (1), 7463. 10.1038/s41598-021-86769-z 33811227 PMC8018957

[B13] Coresh J. Hu J. R. Bello A. K. Feldman H. I. Fogo A. B. Ganji M. R. (2017). Action plan for determining and monitoring the prevalence of chronic kidney disease. Kidney Int. Suppl. 7 (2), 63–70. 10.1016/j.kisu.2017.07.002 PMC6341010 30675421

[B14] Dhillon-Jhattu S. Sprague S. M. (2018). Should phosphate management be limited to the KDIGO/KDOQI guidelines? Seminars dialysis 31 (4), 377–381. 10.1111/sdi.12702 29671909

[B15] Erem S. Razzaque M. S. (2018). Dietary phosphate toxicity: an emerging global health concern. Histochem. Cell Biol. 150 (6), 711–719. 10.1007/s00418-018-1711-8 30159784

[B16] Fishbane S. Block G. A. Loram L. Neylan J. Pergola P. E. Uhlig K. (2017). Effects of ferric citrate in patients with nondialysis-dependent CKD and iron deficiency anemia. J. Am. Soc. Nephrol. JASN 28 (6), 1851–1858. 10.1681/ASN.2016101053 28082519 PMC5461803

[B17] Francis C. Courbon G. Gerber C. Neuburg S. Wang X. Dussold C. (2019). Ferric citrate reduces fibroblast growth factor 23 levels and improves renal and cardiac function in a mouse model of chronic kidney disease. Kidney Int. 96 (6), 1346–1358. 10.1016/j.kint.2019.07.026 31668632 PMC6875640

[B18] Iguchi A. Yamamoto S. Yamazaki M. Tasaki K. Suzuki Y. Kazama J. J. (2018). Effect of ferric citrate hydrate on FGF23 and PTH levels in patients with non-dialysis-dependent chronic kidney disease with normophosphatemia and iron deficiency. Clin. Exp. Nephrol. 22 (4), 789–796. 10.1007/s10157-017-1510-x 29181658

[B19] Iida A. Matsushita M. Ohta T. Yamada T. (2020). Conventional and novel impacts of ferric citrate on iron deficiency anemia and phosphorus metabolism in rats. J. veterinary Med. Sci. 82 (3), 379–386. 10.1292/jvms.19-0641 PMC7118491 31996496

[B20] Inker L. A. Astor B. C. Fox C. H. Isakova T. Lash J. P. Peralta C. A. (2014). KDOQI US commentary on the 2012 KDIGO clinical practice guideline for the evaluation and management of CKD. Am. J. kidney Dis. official J. Natl. Kidney Found. 63 (5), 713–735. 10.1053/j.ajkd.2014.01.416 24647050

[B21] Khairallah P. Isakova T. Asplin J. Hamm L. Dobre M. Rahman M. (2017). Acid load and phosphorus homeostasis in CKD. Am. J. kidney Dis. official J. Natl. Kidney Found. 70 (4), 541–550. 10.1053/j.ajkd.2017.04.022 PMC5804342 28645705

[B22] Lewis J. B. Sika M. Koury M. J. Chuang P. Schulman G. Smith M. T. (2015). Ferric citrate controls phosphorus and delivers iron in patients on dialysis. J. Am. Soc. Nephrol. JASN 26 (2), 493–503. 10.1681/ASN.2014020212 25060056 PMC4310662

[B23] Lin Y. Meng L. Xu X. Du J. Tang Z. Li Y. (2021). The effects of iron citrate on serum fibroblast growth factor-23, cardiac function indicators, and the risk of renal function progression in patients with chronic kidney disease. Chin. Med. J. 56 (09), 975–979.

[B24] Nakagawa Y. Komaba H. (2018). Secondary osteoporosis. Disordered bone metabolism in chronic kidney disease. Clin. calcium 28 (12), 1611–1618. Available at: https://doi.org/clica181216111618. 30487325

[B25] Nakanishi T. Hasuike Y. Nanami M. Yahiro M. Kuragano T. (2016). Novel iron-containing phosphate binders and anemia treatment in CKD: oral iron intake revisited. Nephrol. dialysis, Transplant. official Publ. Eur. Dialysis Transpl. Assoc. - Eur. Ren. Assoc. 31 (10), 1588–1594. 10.1093/ndt/gfv268 26142396

[B26] Nastou D. Fernández-Fernández B. Elewa U. González-Espinoza L. González-Parra E. Sanchez-Niño M. D. (2014). Next-generation phosphate binders: focus on iron-based binders. Drugs 74 (8), 863–877. 10.1007/s40265-014-0224-6 24848754

[B27] Negri A. L. Ureña Torres P. A. (2015). Iron-based phosphate binders: do they offer advantages over currently available phosphate binders? Clin. kidney J. 8 (2), 161–167. 10.1093/ckj/sfu139 25815172 PMC4370297

[B28] Ogata H. Takeshima A. Ito H. (2022). An update on phosphate binders for the treatment of hyperphosphatemia in chronic kidney disease patients on dialysis: a review of safety profiles. Expert Opin. drug Saf. 21, 947–955. 10.1080/14740338.2022.2044472 35180026

[B29] Pai A. B. Jang S. M. Wegrzyn N. (2016). Iron-based phosphate binders--a new element in management of hyperphosphatemia. Expert Opin. drug metabolism Toxicol. 12 (1), 115–127. 10.1517/17425255.2016.1110573 26572591

[B30] Palmer S. C. Gardner S. Tonelli M. Mavridis D. Johnson D. W. Craig J. C. (2016). Phosphate-binding agents in adults with CKD: a network meta-analysis of randomized trials. Am. J. kidney Dis. official J. Natl. Kidney Found. 68 (5), 691–702. 10.1053/j.ajkd.2016.05.015 27461851

[B31] Rivera R. F. Alibrandi M. T. S. Di Lullo L. Fioccari F. (2017). Clinical management of anemia in patients with CKD. G. Ital. Nefrol. organo Uff. della Soc. Ital. Nefrol. 34 (Suppl. 69), 20–35. 28682026

[B32] Scheers N. M. Pereira D. I. A. Faria N. Powell J. J. (2018). Ferric citrate and ferric EDTA but not ferrous sulfate drive amphiregulin-mediated activation of the MAP kinase ERK in gut epithelial cancer cells. Oncotarget 9 (24), 17066–17077. 10.18632/oncotarget.24899 29682205 PMC5908306

[B33] Schumacher S. P. Schurgers L. J. Vervloet M. G. Neradova A. (2019). Influence of pH and phosphate concentration on the phosphate binding capacity of five contemporary binders. An *in vitro* study. Nephrol. Carlt. Vic. 24 (2), 221–226. 10.1111/nep.13245 PMC6585603 29479762

[B34] Segregur D. Flanagan T. Mann J. Moir A. Karlsson E. M. Hoch M. (2019). Impact of acid-reducing agents on gastrointestinal physiology and design of biorelevant dissolution tests to reflect these changes. J. Pharm. Sci. 108 (11), 3461–3477. 10.1016/j.xphs.2019.06.021 31265846

[B35] Shah H. H. Hazzan A. D. Fishbane S. (2015). Novel iron-based phosphate binders in patients with chronic kidney disease. Curr. Opin. Nephrol. Hypertens. 24 (4), 330–335. 10.1097/MNH.0000000000000128 26050119

[B36] Tran L. Batech M. Rhee C. M. Streja E. Kalantar-Zadeh K. Jacobsen S. J. (2016). Serum phosphorus and association with anemia among a large diverse population with and without chronic kidney disease. Nephrol. dialysis, Transplant. official Publ. Eur. Dialysis Transpl. Assoc. - Eur. Ren. Assoc. 31 (4), 636–645. 10.1093/ndt/gfv297 PMC4805130 26254460

[B37] Wang J. Zhang X. Y. Guan Y. F. (2015). Hyperphosphatemia in chronic kidney disease (CKD). Sheng li ke xue jin zhan Prog. physiology 46 (4), 241–244. 26669072

[B38] Womack R. Berru F. Panwar B. Gutiérrez O. M. (2020). Effect of ferric citrate versus ferrous sulfate on iron and phosphate parameters in patients with iron deficiency and CKD: a randomized trial. Clin. J. Am. Soc. Nephrol. CJASN 15 (9), 1251–1258. 10.2215/CJN.15291219 32694162 PMC7480557

[B39] Wu M. Y. Chen Y. C. Lin C. H. Wu Y. C. Tu Y. K. Tarng D. C. (2017). Safety and efficacy of ferric citrate in phosphate reduction and iron supplementation in patients with chronic kidney disease. Oncotarget 8 (63), 107283–107294. 10.18632/oncotarget.21990 29291028 PMC5739813

[B40] Wu-Wong J. R. Chen Y. W. Gaffin R. Hall A. Wong J. T. Xiong J. (2014). VS-501: A NOVEL, NON-ABSORBED, CALCIUM- AND ALUMINUM-FREE, HIGHLY EFFECTIVE PHOSPHATE BINDER DERIVED FROM NATURAL PLANT POLYMER. Pharmacol. Res. Perspect. 2 (3), e00042. 10.1002/prp2.42 25197556 PMC4151863

[B41] Xu Y. Peng H. Ke B. (2017). α-klotho and anemia in patients with chronic kidney disease patients: a new perspective. Exp. Ther. Med. 14 (6), 5691–5695. 10.3892/etm.2017.5287 29250136 PMC5729369

[B42] Yagil Y. Fadem S. Z. Kant K. S. Bhatt U. Sika M. Lewis J. B. (2015). Managing hyperphosphatemia in patients with chronic kidney disease on dialysis with ferric citrate: latest evidence and clinical usefulness. Ther. Adv. chronic Dis. 6 (5), 252–263. 10.1177/2040622315589934 26336594 PMC4549692

[B43] Yokoyama K. Hirakata H. Akiba T. Fukagawa M. Nakayama M. Sawada K. (2014). Ferric citrate hydrate for the treatment of hyperphosphatemia in nondialysis-dependent CKD. Clin. J. Am. Soc. Nephrol. CJASN 9 (3), 543–552. 10.2215/CJN.05170513 24408120 PMC3944759

