# Supplementary material for: Ferric citrate for the treatment of hyperphosphatemia and iron deficiency anaemia in patients with NDD-CKD: a systematic review and meta-analysis
Source: Front Pharmacol. 2024 Mar 7;15:1285012. doi: 10.3389/fphar.2024.1285012 (PMC10955115; doi:10.3389/fphar.2024.1285012)
Supplement: Supplementary file 1 [file Table1.DOCX]

# Supplementary material

# Search strategy

PubMed: 111

#3 AND #6

#6 #4 OR #5 204732

#5 (((Kidney Insufficiencies, Chronic[All Fields) OR (chronic kidney disease[All Fields)) OR (Chronic Renal Insufficiencies[All Fields)) OR (Renal Disease, Chronic[All Fields)

#4 "Chronic kidney disease"[Mesh]

#3 #1 OR #2 1828

#2 (((((JTT-751[All Fields]) OR (iron(III) citrate[All Fields])) OR (ferric citrate hydrate[All Fields])) OR (ferric citrate anhydrous[All Fields])) OR (ferric-citric acid[All Fields])

#1 "Ferric citrate"[Mesh]

Web of Science:136

#1 AND #2

#1 TS=((((Kidney Insufficiencies, Chronic) OR (chronic kidney disease)) OR (Chronic Renal Insufficiencies)) OR (Renal Disease, Chronic)) 99414

#2 TS=((((((JTT-751) OR (ferric citrate)) OR (iron(III) citrate)) OR (ferric citrate hydrate)) OR (ferric citrate anhydrous)) OR (ferric-citric acid)) 1534

Scopus:103

#1 AND #2 AND #3

#1 TITLE-ABS-KEY(((Kidney Insufficiencies, Chronic) OR (chronic kidney disease)) OR (Chronic Renal Insufficiencies)) OR (Renal Disease, Chronic) 46371

#2 TITLE-ABS-KEY(((((JTT-751) OR (ferric citrate)) OR (iron(III) citrate)) OR (ferric citrate hydrate)) OR (ferric citrate anhydrous)) OR (ferric-citric acid) 967

EBSCO:202

(S1 AND S2)

#1 TX (((Kidney Insufficiencies, Chronic) OR (chronic kidney disease)) OR (Chronic Renal Insufficiencies)) OR (Renal Disease, Chronic) 57923

#2 TX (((((JTT-751) OR (ferric citrate)) OR (iron(III) citrate)) OR (ferric citrate hydrate)) OR (ferric citrate anhydrous)) OR (ferric-citric acid) 1426

Cochrane:72

#1 AND #2

#1 (((Kidney Insufficiencies, Chronic) OR (chronic kidney disease)) OR (Chronic Renal Insufficiencies)) OR (Renal Disease, Chronic) in Title Abstract Keyword - (Word variations have been searched) 71

#2 (((((JTT-751) OR (ferric citrate)) OR (iron(III) citrate)) OR (ferric citrate hydrate)) OR (ferric citrate anhydrous)) OR (ferric-citric acid) in Title Abstract Keyword - (Word variations have been searched) 170

Embase:240

#1 AND #2

#1 (((Kidney Insufficiencies, Chronic) OR (chronic kidney disease)) OR (Chronic Renal Insufficiencies)) OR (Renal Disease, Chronic) 3745781

#2 (((JTT-751) OR (ferric citrate)) OR (iron III citrate)) OR (ferric citrate hydrate) OR (ferric citrate anhydrous) OR (ferric-citric acid) in All Text - (Word variations have been searched) 3752

CNKI:52

(subject =ferric citrate ) and (subject = chronic kidney disease) 52

Wang Fang Date:5

All: (ferric citrate) and all: (chronic kidney disease)) 5

VIP:1

((any field = ferric citrate) and any field =chronic kidney disease ) 1
